# Effects of Flipped Classrooms on the Academic Achievements, Individualised Education Plan Competencies and Quality of Related Preparation of Pre-Service Teachers

**DOI:** 10.3390/bs15040438

**Published:** 2025-03-28

**Authors:** Hande Durmuşoğlu, Mukaddes Sakalli Demirok

**Affiliations:** Department of Special Education, Near East University, 99138 Nicosia, Cyprus; mukaddes.sakalli@neu.edu.tr

**Keywords:** flipped classroom, educational technology, special education, pre-service teachers, individualised education programme

## Abstract

Flipped classrooms are a pedagogically appropriate approach that supports inclusive education by increasing in-class practise time, including active learning activities. They can support permanence by reinforcing many courses taught theoretically in universities. In this respect, flipped classrooms can become an important advantage in the training of special education teachers. In this study, we aimed to examine the association between the flipped classroom on pre-service special education teachers’ academic achievement, Individualised Education Plan (IEP) competencies and IEP quality and to determine pre-service teachers’ views on the flipped IEP course. In our research, we investigated a sample of 66 s-year pre-service teachers, 33 of whom were randomised into an experimental group, and 33 into a control group. Participants were randomly assigned to the experimental and control groups. As a result of this study, it was revealed that the flipped IEP course had a statistically significant association with the quality of the IEP prepared by the pre-service teachers, that the information they gained in the IEP course was permanent, that the subjects were more understandable, that it provided enjoyable learning opportunities based on practise, and that it increased classroom interaction.

## 1. Introduction

Flipped classrooms are emerging as a practical and innovative pedagogical approach in higher education and are spreading worldwide with increasing impact (Baig & Yadegaridehkordi, 2023; Tucker, 2012). In the digital age, technology is integral to education. In contrast to the traditional teaching approach, theories and models based on flipped learning position students at the centre and ensure their active participation in the learning process (Freeman et al., 2014). Through flipped learning, students actively engage in learning by doing and experiencing (Toykok et al., 2021). The flipped classroom model fosters self-directed learning and encourages students to take responsibility for their education (Fulton, 2012). This paradigm shift also challenges academics to adapt to new teaching roles. A well-designed flipped classroom can enable students to spend meaningful time sitting quietly in a classroom on campus, listening, observing and taking notes and practising with active participation (Reidsema et al., 2017). Research on flipped classrooms in higher education demonstrated that they positively contribute to learning outcomes but raise challenges and concerns and call for evidence-based practise (Baig & Yadegaridehkordi, 2023; Bishop & Verleger, 2013). Despite these challenges, flipped classrooms offer significant pedagogical innovation.

However, while flipped learning presents numerous advantages, it is important to acknowledge that these benefits do not apply uniformly across all student demographics. Challenges related to digital accessibility, as well as differing levels of technological proficiency, can disproportionately affect certain student groups, including those from lower socio-economic backgrounds (Altemueller & Lindquist, 2017). Furthermore, while flipped learning is generally framed as an active learning strategy, there remains a gap in research concerning whether this model truly benefits special education teacher training in the same way it benefits general education (Gargiulo & Metcalf, 2017). This necessitates a more focused investigation into the specific pedagogical advantages of flipped learning for pre-service special education teachers, beyond the general benefits of active learning principles.

Teacher education programmes play a pivotal role in influencing education systems and the academic achievement of students. These programmes aim to develop the professional knowledge, skills and attitudes of prospective teachers, as well as train them to be effective and well-equipped educators (Darling-Hammond, 2006). A well-designed training programme for special education teachers should include theoretical knowledge and practical application (Gargiulo & Metcalf, 2017). Training high-quality special education teachers is the main goal of these programmes, especially as the number of students with special needs continues to rise (Friend & Bursuck, 2014). As such, training special education teachers using innovative models such as flipped learning could enhance the acquisition of both pedagogical knowledge and practical skills (Brewer & Movahedazarhouligh, 2018). This model allows students to learn content at home through online materials and engage in hands-on activities during class (Bergmann & Sams, 2012). It provides pre-service teachers with opportunities to experience and practise various pedagogical strategies in a more flexible, student-centred environment. Although flipped learning has been successfully implemented across various educational levels, its specific advantages for special education training, especially in terms of developing tailored teaching strategies, are yet to be fully explored (Friend & Bursuck, 2014; Goodwin & Miller, 2013).

Research by Chuang et al. (2018) shows that the flipped classroom method enhances student engagement across various subjects by combining classroom activities with practical experiences. This approach supports individual learning paces and enhances comprehension of complex subjects by allowing pre-service teachers to revisit lecture videos (Bishop & Verleger, 2013). It fosters collaborative learning environments, promoting teamwork during class time. Pre-learning materials enable active classroom tasks like discussions, problem-solving and project work.

Pre-service teachers also develop digital proficiency through creating and utilising online course content (Gannod et al., 2008). This model facilitates meaningful interactions between pre-service teachers and students. Additionally, student-centred teaching strategies help teachers better understand student needs (Davies et al., 2013). Although initially implemented in high schools, flipped classrooms have been widely adopted across educational levels, with consistent positive outcomes in secondary and higher education (Altemueller & Lindquist, 2017).

Instructors who plan and execute their educational content well can improve student results and enthusiasm in flipped learning environments. Teachers need to spend extra time developing course content before the class using video resources or alternative materials (Bergmann & Sams, 2012). Students need suitable technology at home for this approach which creates unfairness in education and restricts their ability to access educational resources. The issue of equitable access to technology for students in socio-economically disadvantaged regions must be addressed, as it poses a significant challenge to the widespread adoption of flipped learning. Students often need outside encouragement to study at home independently while keeping their course materials ready for work. The main problem students face is not having access to the lecture videos from outside their classroom. Scholars emphasise that lecture videos should be brief and interactive (Karaoğlan Yılmaz & Yılmaz, 2019). Preparing the course content in advance and engaging in interactive activities in the classroom require discipline. A number of studies propose that the flipped classroom model does not significantly improve student performance or is less effective than traditional teaching methods in certain cases (Bishop & Verleger, 2013). Inadequate infrastructure for the use of technology across geographical regions, income differences between individuals and social and cultural inequalities stand out as problems to be considered.

### 1.1. Innovative Pedagogical Approaches for the Training of Special Education Teachers

The flipped classroom model can be a new way to train future teachers in special education (Brewer & Movahedazarhouligh, 2018). The main purpose of training programmes for special education teachers is to qualify and equip them to respond to the educational needs of students with special needs (Brownell et al., 2010). Teacher candidates should acquire basic knowledge and skills in the field of special education and learn and apply inclusive education strategies to meet the needs of diverse student groups. With technological developments, scholars discussed the use of digital tools in special education and how these tools can improve student achievement (Ok & Rao, 2019). Future special education teachers should possess skills in using digital tools and supporting technologies to teach effectively in technology-equipped classrooms. Special education teachers should develop social and emotional learning skills and create positive learning environments using these skills (Jennings & Greenberg, 2009).

Future special education teachers should be open to continuous professional development throughout their career and follow new teaching strategies, technologies and research findings.They should have the cultural competence and awareness skills to meet the needs of students from different cultural backgrounds (Gay, 2018). In addition, learning evidence-based practises and using these practises in their classrooms are crucial (Cook & Odom, 2013). Another important skill is collaboration and teamwork. Teachers should be capable of collaborating effectively with colleagues, other teachers, field experts and families. Today, the gap between theory and practise in the field of special education remains, and discussion on the search for strategies for closing this gap continues. Studies propose that special education field courses, which are theoretically conducted except for teaching practise courses, should be increased by prioritising models that emphasise practise. Research indicates that pre-service teachers require more practical experience in field courses. Although the content of teacher training programmes for special education is different, the majority of the components are similar. One of the problems identified is that teachers who lack experience in the field perceive disconnection and incompatibility between knowledge gained in programmes and current practises (Bay & Parker-Katz, 2009).

The shortage of qualified personnel and the lack of special education teachers with sufficient practical experience continue to be amongst the most important challenges in the field. These challenges make new special educators more vulnerable than others and may result in more teachers leaving the profession after a short period of time. Therefore, a need emerges for innovative approaches that emphasise the implementation of the existing programmes to train more effective and qualified special education teachers (Brewer & Movahedazarhouligh, 2018).

### 1.2. Individualised Education Programme Course in Education Undergraduate Programmes for Special Education Teachers

An Individualised Education Programme (IEP) is a dynamic road map that focuses on students with special needs; aims to maximise student performance; intends to meet the needs of students, families and teachers; determines the frequency, duration and type of appropriate education and support services; is prepared and implemented in writing by persons or teams assigned through legal legislation; and makes necessary changes according to student progress (Artar & Ergenekon, 2020).

One of the most important objectives of the IEP course content in undergraduate programmes for special education teachers is that teachers can prepare a functional, effective and high-quality IEP in accordance with the existing performance level of students with special needs. Teachers perform a crucial function in determining, creating and evaluating the behaviour of students. For example, in Turkey, teachers whose duties in the development of IEP are clearly stated in Decree Law No. 573 are required to prepare new goals and implement education programmes according to the development of the individual (İlik & Sarı, 2018). Discussions on the effective implementation of IEP processes (Giangreco & Broer, 2005), efficacy of the prepared IEP or the appropriateness of goals have led to the concept of IEP quality (Gibb & Dyches, 2015). Therefore, enhancing the IEP competencies of teachers and evaluating the quality of the prepared IEPs are important aspects.

Previous studies demonstrate that teachers report difficulty in preparing IEPs and insufficient knowledge of the content of IEPs. Moreover, the majority of special education teachers and other branch teachers do not prepare IEPs and encounter difficulty in writing objectives appropriate to performance and in determining and using teaching methods (Çıkılı et al., 2020; İlik & Sarı, 2018). The IEP course is conducted theoretically in undergraduate programmes, but research results indicate that pre-service special education teachers need more practical experience. This study looks into how using flipped classrooms can help overcome important issues in special education teacher education. Special education teachers struggle to find qualified job candidates because these professionals lack real-world teaching skills and proper training methods. The flipped classroom helps pre-service teachers study course materials at home first and use class time for practical and hands-on learning activities. This teaching technique helps students master difficult material while teaching them the digital skills they need for current classroom technology.

This study recommends stronger development methods for Individual Education Plans that form the core of special education. The active-learning approach integrated with technology in flipped classrooms helps develop skilled teachers and better IEPs through improved practises. Our research investigates special education’s underexplored use of flipped learning to demonstrate ways modern teacher training can benefit from fresh educational approaches.

This study assessed whether pre-service special education teachers showed improved academic performance and enhanced their Individualised Education Programme skills through our flipped course. This research explored the questions related to achieving our main objective.

1. Is there a statistically significant difference between the pre- and post-test scores of the experimental and control groups in an IEP Academic Achievement Test?
2. Is there a statistically significant difference between the pre- and post-test scores of students in the experimental and control groups on the IEP Efficacy Scale?
3. Is there a statistically significant difference between students’ scores on the different sub-dimensions of the IEP Quality Assessment Scale?
4. Is there a statistically significant correlation between students’ scores on the IEP Quality Assessment Scale and their post-test scores on the IEP Academic Achievement Test and IEP Efficacy Scale?

## 2. Materials and Method

The research used an experimental design with one test group taking assessments both before and after the research period. The design tracks how the intervention works by examining the same research group at two distinct points in time. By testing participants twice, this research approach enables researchers to see how the dependent variable responds through comparing their test scores at both time points (Creswell, 2014). The study group’s dependent variable responses were collected using the same evaluating tools before the intervention (pre-test) and following the intervention (post-test).

We conducted a pre-test to measure how much the study group knew about the independent variable before we started the experiment. We used the post-test to assess the effectiveness of the intervention by tracking changes in the dependent variable. To obtain valid findings, we employed experimental research techniques, including randomisation and control groups, to minimise confounding variables and ensure reliable, measurable results, as recommended by Creswell (2014).

### 2.1. Participant

The research took place at a private university’s Educational Sciences Faculty where 66 s-year special education teaching students served as participants. The criterion sampling method was used as our purposeful selection tool to recruit second-year special education students who align with our study goals and can help answer our research questions (Büyüköztürk et al., 2020). The criteria for selection were as follows: (1) being a second-year special education student, (2) being new to creating Individualised Education Plans (IEPs), and (3) being new to implementing flipped classroom teaching strategies.

Second-year students served as participants due to their educational transition from learning theories to gaining practical expertise. Students should possess comprehensive basic understanding of their subject while showing interest in adopting modern teaching methods at this level. During this time, students can embrace the flipped classroom approach objectively to assess its impact because they enter the experiment with no preconceived ideas (Creswell, 2014).

The participants were divided into two groups through a randomisation process. A random draw was employed, using a computer-generated random number sequence, to allocate the 66 students into the experimental and control groups for the experiment. The experimental group learned by following the flipped classroom strategy through online materials before working on homework tasks independently. Students engaged in application-oriented activities during their direct in-person meetings, which included problem-solving and collaborative tasks designed to promote deeper understanding and practical application of the content. In contrast, the control group received traditional instruction, with teaching methods remaining unchanged. They participated in regular lectures and completed individual homework tasks, which were less application-oriented compared to the flipped classroom group. This approach ensured a clear distinction between the two groups’ educational experiences.

### 2.2. Course Design and Procedure

During the spring 2023–2024 academic year, we ran the flipped IEP course for 10 weeks at a university. This study was designed in three phases: we made our strategy, put our plan into action and checked to verify success. Our team built a step-by-step approach that let us use the flipped model correctly and get students more involved with their lessons.

During planning, the researcher examined both IEP course learning goals and student technology access to the internet. We chose digital materials for teaching content while creating the video materials that would be shared with students. The researcher designed classroom activities to match video content by turning theoretical knowledge into practical exercises during live sessions. The approach was meticulously planned to ensure the flipped classroom model remained practical for this course.

We used the LMS and Genially platforms to support students during the flipped learning implementation. Every student had full LMS access and internet connectivity for active participation in our course materials. We selected Genially because it helps students create interactive slides for their coursework through engaging multimedia presentations. IT Support helped students and instructors solve their technical issues during the program. Classroom teaching used an active learning approach through methods such as brainstorming, concept mapping, question rounds and scenario error detection while adding further techniques such as fishbone diagrams, KWL sessions, “last word” activities and question bank construction with case studies and group assignments. Students used these activities to learn how to better analyse information as well as work together while deepening their understanding of the class topics.

During the evaluation phase, we gave students immediate feedback about their work through process-oriented assessments to find their learning obstacles. We evaluated student learning by combining self-assessment tools with peer reviews and projects while using rating scales and digital tools for complete assessment. Our choice of testing methods reflects the active learning design of flipped classrooms while offering students both personal and collective performance evaluation.

### 2.3. Data Collection and Analysis

Quantitative data were collected using the IEP Academic Achievement Test, IEP Efficacy Scale, and IEP Quality Assessment Scale. The data were analysed using SPSS 27.0, and the validity and reliability of the IEP Academic Achievement Test were thoroughly examined, leading to the development of its final form.

The initial version of the IEP Academic Achievement Test included 45 questions. These questions were analysed based on item difficulty (Pj) and item discrimination (Rjx) indices. Following the analysis, questions that were categorised as very easy (Pj > 0.80), very difficult (Pj < 0.20), or those with low discrimination (Rjx < 0.20) or negative discrimination indices were removed from the test. As a result, questions 9, 10, 12, 14, 16, 17, 19, 20, 21, 31, 33, 34, 42 and 45 were retained, while the others were excluded. The final version of the test was refined to include a total of 14 questions.

The validity and reliability analysis of the test was conducted with third-year students, who were not part of the main study sample, to ensure that the test was free from bias in the final implementation. After the refinement process, the final version of the test was administered to second-year students as part of the main study. This approach ensured that the validation process did not interfere with the results of the experimental and control groups, maintaining the objectivity and credibility of the findings.

The validity of the test was ensured through several measures. Content validity was established by having the test items reviewed by subject matter experts to ensure their relevance and alignment with the learning objectives of the course. Construct validity was assessed by examining the relationships between test scores and other established academic achievement measures. Criterion-related validity was also considered by comparing the test scores with real-world performance outcomes in the IEP course.

The reliability of the test was assessed using multiple statistical methods. The Cronbach’s alpha coefficient was calculated as 0.741, indicating a high level of internal consistency and strong coherence among the items. A Cronbach’s alpha value of 0.70 or higher is considered acceptable for psychological tests (Nunnally, 1978). The split-half reliability analysis yielded a split-half correlation coefficient of 0.720 and a Spearman–Brown correction coefficient of 0.713. Additionally, the Kuder–Richardson 20 (KR20) and Kuder–Richardson 21 (KR21) coefficients were calculated as 0.803 and 0.808, respectively, demonstrating that the test items are homogeneous and the measurement error is minimal. Values above 0.70 for KR20 and KR21 indicate sufficient reliability (Kuder & Richardson, 1937).

The 14 questions in the final version of the test displayed balanced levels of difficulty and discrimination. Regarding difficulty levels, questions 12 and 42 were classified as “moderate”, while questions 9, 16, 17, 19, 20, 21, 31, 33, 34 and 45 were classified as “easy”. In terms of discrimination indices, questions 21 and 34 were found to be “very suitable”, while questions 12, 19, 20, 33, 42 and 45 were classified as “suitable”. Questions 9, 10, 14, 16, 17 and 31 were categorised as “partially suitable” in terms of discrimination indices.

In conclusion, the final version of the IEP Academic Achievement Test, consisting of 14 questions, was determined to be a valid and reliable tool for measuring the knowledge levels of pre-service teachers. The final test served as a primary instrument for evaluating academic achievement in the IEP course. This process successfully supported the objective of assessing both theoretical and practical learning outcomes in the course. Demographic variables such as age, gender and level of university education were compared between the two groups using statistical methods. A normality test was conducted prior to the analysis, and the data were found to follow a normal distribution. Accordingly, an independent samples *t*-test was performed for gender and level of university education, while a one-way ANOVA was used to analyse age differences. Additionally, an independent samples *t*-test was also used to compare the control and experimental groups.

## 3. Results

The socio-demographic characteristics of the participants in the control and experimental groups are presented in Table 1.

Demographic variables such as age, gender and level of university education were compared between the two groups and analysed using statistical methods. Table 1 presents the comparison of demographic variables between the experimental and control groups. *p*-values indicate statistical significance levels.

Table 2 presents the comparison of changes in pre- and post-test scores of the control and experimental groups in the Individualised Education Plan Academic Achievement Test.

The analysis compares the differences between the pre- and post-test scores of the two groups (rather than comparing only post-test scores). The aim is to assess the impact of the flipped classroom model applied to the experimental group versus the traditional teaching method used with the control group on students’ academic performance. 

The difference in post-test scores between the experimental and control groups was statistically significant (*t* = 11.797, *p* < 0.05). The effect size (*η*^2^ = 0.158) indicates a moderate impact of the flipped classroom model on the IEP Academic Achievement Test results.

In conclusion, the findings in Table 2 reveal that the flipped classroom model is an effective method for enhancing students’ academic achievement in the IEP course. The results suggest that this model allows students to develop a deeper understanding of Individualised Education Plans and achieve learning outcomes more effectively.

Table 2 presents the comparison of pre-test and post-test scores of the control and experimental groups based on the Individualised Education Plan (IEP) Competency Scale. The analysis examines the sub-dimensions of IEP preparation, creation, family participation, implementation and evaluation.

Although slight increases were observed in the post-test mean scores across all sub-dimensions for both groups, none of these differences were statistically significant (*p* > 0.05). Similarly, the overall IEP Competency Scale showed minor improvements, but no significant effect was found. These results suggest that the flipped classroom model did not lead to a statistically significant improvement in IEP competency compared to the traditional method. The *p*-values reported in Table 2 represent the differences between pre-test and post-test scores. Additionally, these differences were compared between the experimental and control groups to assess the impact of the flipped classroom model on IEP competency.

Finally, for the overall IEP Competency Scale, the control group’s pre-test mean score was 110.73 (s = 15.75) and the post-test mean score increased to 118.21 (s = 14.32). For the experimental group, the pre-test mean was 110.73 (s = 21.95) and the post-test mean was 119.85 (s = 10.86). No statistically significant difference was observed in this dimension either (*t* = 0.270, *p* = 0.605, *η*^2^ = 0.004).

In this study, the consistency and homogeneity of the experts’ evaluations of students’ scores were analysed. The experts, professionals in education and assessment, were responsible for evaluating the quality of the IEP components. It is important to note that assessors were not blinded to the group membership of the students they graded, which may have influenced the grading process.

The analysis of Table 3 indicates that there were no statistically significant differences between students’ scores across the Instructional Components and Complementary Components sub-dimensions (*p* > 0.05). The results suggest that expert evaluations remained consistent and homogeneous, with no notable variation among scorers.

For the overall IEP Quality Assessment Scale, Scorer 1’s mean score was 23.88 (s = 4.48), Scorer 2’s mean score was 23.67 (s = 4.51), and Scorer 3’s mean score was 23.45 (s = 4.50). The minimum score was 10, and the maximum score was 29. The ANOVA results show that there was no statistically significant difference in the overall scores between the students (F = 0.073, *p* = 0.929).

Table 4 presents the correlations between the post-test scores of the experimental group’s students on the Individualised Education Plan (IEP) Quality Assessment Scale, the IEP Academic Achievement Test and the IEP Efficacy Scale. This analysis examines the relationships between students’ scores on the IEP Quality Assessment Scale and their academic achievement and competency levels.

The findings indicate that no statistically significant correlations were found between the IEP Quality Assessment Scale and either the IEP Academic Achievement Test or the IEP Efficacy Scale (*p* > 0.05). This suggests that students’ performance on the Instructional Components, Complementary Components, and overall IEP Quality Assessment Scale is not strongly associated with their academic achievement or competency levels.

In conclusion, the findings from Table 4 indicate that there is no statistically significant relationship between the IEP Quality Assessment Scale and the students’ academic achievement or competency levels. This suggests that students’ success on the BEP evaluations is not directly linked to traditional academic measures, and the two assessment tools likely evaluate different sets of skills and knowledge.

## 4. Discussion

The findings indicate that students in the experimental group who used flipped classroom methods showed better learning results compared to the control group (*t* = 11.797, *p* < 0.05). Furthermore, the experiment demonstrated that higher academic gains were achieved by the experimental group compared to the control group. These results suggest that the flipped classroom model not only helps students learn more effectively but also provides an efficient learning option for personalised education. This finding aligns with the research conducted by Bergmann and Sams (2012), which highlights how the flipped classroom approach shifts classroom hours from self-study to interactive learning activities while allowing students to learn content at home. Additionally, this training model plays a crucial role in helping special education teachers develop practical teaching skills, as it exposes them to different strategies during their education programme.

Research shows that using the flipped classroom approach improves student learning and raises grades in teacher training programmes (Baig & Yadegaridehkordi, 2023; Fulton, 2012). Active involvement in lessons plus flexible self-paced learning combined with better class discussions helped students achieve better results according to Bishop and Verleger (2013) and Abeysekera and Dawson (2015). Pre-service teachers learn basic teaching methods by viewing pre-created video lectures which they confirm through physical practise in the classroom (O’Flaherty & Phillips, 2015).

Academic studies demonstrate that pre-service teachers both perform well and feel happy while using this instructional method (Strayer, 2012). Standards of critical thinking and problem solving improve when students learn through this teaching model (Lo & Hew, 2017). Our study results (*η*^2^ = 0.158; *p* < 0.05) ** indicate a statistically significant association of the method; however, a more detailed interpretation of its practical implications is essential. Effect size (*η*^2^ = 0.158) represents a moderate influence (Cohen, 1988), which implies that the flipped classroom approach can contribute meaningfully to learning outcomes but may not be universally applicable. However, the effectiveness of this method varies based on factors such as students’ prior knowledge, motivation levels and teaching strategies (Strelan et al., 2020).

Given the moderate effect size, further research should explore its applicability and impact in broader educational contexts, particularly within social sciences and the arts, to determine whether similar benefits can be observed across different disciplines (Karabulut-Ilgu et al., 2018). This would provide a more comprehensive understanding of its pedagogical value beyond STEM disciplines and inform best practises for its implementation in diverse learning environments (Bond, 2020).

The results of students’ IEP Competency Scale remained unchanged when the flipped classroom teaching strategy was applied. Although the experimental group exhibited improvement, statistical analysis indicated that the growth was not statistically significant (as confirmed by the *p*-value analysis). While the teaching strategy led to an enhancement in test scores, it did not produce a substantial change in students’ self-assessment of their competence levels. This lack of significance may be attributed to several factors, such as a small sample size, small effect size, or high variance. While the teaching strategy enhanced test scores, it is not possible to infer a causal relationship between flipped classroom use and test score improvements based solely on this study.

Causal inference requires careful consideration of study design, control for confounding variables and replication across diverse contexts (Shadish et al., 2002). Competency perceptions are typically influenced by individual experiences, self-efficacy levels and social support systems (Bandura, 1997; Zimmerman, 2000). In the absence of longitudinal data, conclusions regarding causality remain speculative. However, the experimental manipulation in this study—where participants were randomly assigned to either the flipped classroom or traditional teaching method—allows for a clearer evaluation of its impact. The findings indicate an increase in the academic achievement and IEP competencies of the pre-service teachers.

The examination of the relationship between the quality of the Individualised Education Programme (IEP) and academic achievement is an important research topic in the field of special education. The existing literature indicates inconsistent and sometimes statistically non-significant relationships between these two variables. For example, in a study conducted by Aksoy and Alan (2019), it was noted that the overall quality of IEPs developed in special education schools was low, and despite the large number of IEPs, teachers faced difficulties in preparing them. This highlights the importance of providing training to teachers to improve the quality of IEPs. Additionally, a study by Alan and Aksoy (2023) found that the quality of IEPs decreased as the school level increased, and that teachers’ professional experience had an effect on IEP quality.

In light of these findings, it can be said that the relationship between IEP quality and academic achievement is complex and multifactorial. Teachers’ knowledge and skills in preparing and implementing IEPs are only one of many factors affecting students’ academic success. Therefore, future research should explore strategies to enhance IEP quality and teacher competencies and analyse the impact of these factors on academic achievement in more detail. This result was interpreted as striking in terms of revealing the difference between theory and practise.

Moreover, pre-service teachers need more practical experience in their field courses. It has been emphasised that long-term experiences and positive feedback are necessary for students to demonstrate a significant increase in their competency perceptions (Schunk, 2012). Additionally, instructional strategies aimed at enhancing competency perceptions should support student-centred approaches and provide a continuous self-assessment mechanism throughout learning processes (Zimmerman & Schunk, 2011). In this context, the flipped classroom model may not suffice as a standalone method due to its limited impact on competency perceptions.

To strengthen the broader claims regarding its effectiveness in teacher education, further qualitative insights and longitudinal studies are needed to explore how sustained exposure to flipped learning impacts teaching competency development over time (Kurt, 2017; Zainuddin & Perera, 2019). Additionally, incorporating emotional support mechanisms and collaborative learning models in education may yield more positive results (Huang & Lajoie, 2023), particularly in fostering self-efficacy and pedagogical confidence among future educators (Karabulut-Ilgu et al., 2018).

No statistically significant difference was found among evaluators regarding the sub-dimensions and overall evaluation scores of the IEP Quality Assessment Scale. This suggests that the scale can be applied homogeneously and serves as a reliable assessment tool in different contexts. The applicability and reliability of the scale are important for standardising Individualised Education Plans (Reynolds & Teddlie, 2002). However, the evaluation of educational quality requires not only measurement tools but also process-oriented assessment methods. It is recommended that educators and experts establish a continuous feedback loop during the implementation of Individualised Education Plans to enhance quality (Darling-Hammond, 2010).

Correlation analyses between the IEP Quality Assessment Scale, Academic Achievement Test and Competency Scale revealed no statistically significant relationship among these variables. This result indicates that tools used to improve the quality of learning through Individualised Education Plans are not directly associated with traditional academic achievement criteria. This demonstrates that quality measurements in education focus more on assessing pedagogical processes tailored to students’ individual needs (Guskey, 2000).

### Limitations and Future Research

The analyses conducted in this study, involving a limited sample group within a single educational context, restrict the generalisability of the findings. A more detailed discussion of practical implications for educators is necessary, especially regarding the challenges and benefits of implementing the flipped classroom model in different teaching environments. Additionally, the flipped classroom model was implemented only over a short-term period, which hinders the full evaluation of its long-term effects. Longitudinal studies are recommended to assess the sustained impact of flipped learning on academic achievement and perceived competency. It should be noted that changes in students’ competency perceptions typically require longer-term implementations and continuity (Schunk, 2012). Future research should consider employing objective measures, such as direct observation or performance-based assessments, to provide more reliable data. In this study, self-reported measures included the IEP Efficacy Scale and the IEP Quality Assessment Scale, both of which relied on participants’ subjective evaluations. While these measures offer valuable insights into participants’ perceptions, they may be influenced by factors such as self-perception biases or social desirability. These factors should be considered when interpreting the findings.

Furthermore, the validity of the measurement tools used in this study has not been tested in different cultural and educational contexts. Expanding research to include cross-cultural comparisons would allow for a more comprehensive understanding of the flipped classroom model’s effectiveness across diverse learning environments. Future research should investigate the effects on various age groups, disciplines and cultural settings. In addition, external factors, such as socio-economic background or institutional support, might have impacted the outcomes, and these variables warrant further exploration. For instance, the impact of socio-economic factors on educational outcomes in flipped classrooms has been shown to be significant.

The integration of the flipped classroom model not only into Individualised Education Plans but also into other pedagogical approaches could be evaluated. This could include exploring hybrid models that combine flipped learning with traditional teaching methods, which may enhance engagement and cater to different learning styles. This could help identify the contexts in which this model is most effective or where adjustments might be necessary. In conclusion, the flipped classroom model can be used to transform higher education classrooms into dynamic, interactive learning environments in which educators guide the students. The power of flipped learning is related not only to the videos prepared in accordance with the course content but also to the fact that the designed course content changes the nature of learning, which places learning and students at the centre of pedagogy. It can be an alternative method for supporting pre-service special education teachers through new pedagogical approaches.

Finally, the lack of a significant relationship between quality and achievement underscores the need to comprehensively reconsider these assessment tools. Future research should investigate whether alternative methods of assessment, such as peer reviews or project-based evaluations, might yield a clearer picture of student success. Mixed-methods research is suggested to gain a deeper understanding of educational processes (Creswell & Plano Clark, 2017).

## 5. Conclusions

This study has revealed the effects of innovative approaches, such as the flipped classroom model, in the evaluation of Individualised Education Plans, demonstrating significant impacts on academic achievement. However, more limited results were obtained in terms of competency perceptions and quality assessment.

The findings emphasise the importance of Individualised Education Plans in teaching processes. Nevertheless, the use of diverse assessment tools and the examination of the long-term effects of these processes may provide more robust results. Educational policies and practises should be restructured to promote not only academic achievement but also processes that support individuals’ personal development. The ongoing debate in the literature on the gap between theory and practise in teacher education once again points to the importance of practise and pedagogical approaches that emphasise practise. The current study makes important contributions to the training of qualified and well-equipped prospective teachers. Thus, it points to a need for additional comprehensive research on this subject and research findings that comprehensively investigate the effects of the flipped classroom model on pre-service special education teachers and their experiences with this model.

Technology-based and active learning-based models can contribute to the training of qualified teachers. Preparation of quality IEPs can meet the basic needs of institutions, individuals with special needs and families regarding quality programs. The flipped classroom model can be included in teacher training programmes as an important tool in improving the pedagogical skills and use of technology of teacher candidates. The flipped classroom model may have the power to transform higher education classrooms into dynamic, interactive learning environments where the educator guides the students.

## Figures and Tables

**Table 1:** Socio-demographic characteristics of the experimental and control groups.

	Control		Experimental		Total		X2	*p*
	n	%	n	%	n	%		
Age								
18–24	13	39.39	11	33.33	24	36.36		
25–34	19	57.58	20	60.61	39	59.09	0.526	0.769
35–44	1	3.03	2	6.06	3	4.55		
Gender								
Female	11	33.33	14	42.42	25	37.88	0.580	0.447
Male	22	66.67	19	57.58	41	62.12		
Level of University Education								
1st university	15	45.45	17	51.52	32	48.48	0.243	0.622
2nd university	18	54.55	16	48.48	34	51.52		

**Table 2:** Comparison of pre-test and post-test score changes between control and experimental groups in the Individualised Education Plan Academic Achievement Test using *t*-test.

	Group	n	Pre-Test		Post-Test		Difference	*t*	*p*	*η* ^2^
			$\bar{x}$	s	$\bar{x}$	s				
**IEP** **Academic Achievement Test**	Control	33	8.79	2.36	11.94	1.62	3.15	11.797	0.001 *	0.158
	Experiment	33	9.09	2.20	13.06	0.86	3.97			
Before IEP Preparation	Control	33	24.33	3.76	26.36	3.00	2.03	2.621	0.110	0.040
	Experiment	33	24.42	4.21	27.58	3.03	3.16			
IEP Preparation Phase	Control	33	24.76	5.14	25.03	5.95	0.27	0.446	0.507	0.007
	Experiment	33	25.27	5.93	25.82	4.39	0.55			
Family Participation in the IEP	Control	33	16.42	2.62	17.09	2.74	0.67	0.949	0.334	0.015
	Experiment	33	16.00	3.61	17.67	2.19	1.67			
Implementation of the IEP and Evolution	Control	33	45.21	7.69	49.73	6.59	4.52	0.417	0.521	0.007
	Experiment	33	45.03	10.56	48.79	5.20	3.76			
For Teachers IEP Efficiency Scale	Control	33	110.73	15.75	118.21	14.32	7.48	0.270	0.605	0.004
	Experiment	33	110.73	21.95	119.85	10.86	9.12			

* p < 0.05.

**Table 3:** Comparison of experts’ scores on the Instructional and Complementary Components of the IEP Quality Evaluation Scale.

	Scorer	n	$\bar{x}$	s	Min	Max	F	*p*
IEP Instructional Components	Scorer 1	33	10.73	2.80	3	14	0.065	0.937
	Scorer 2	33	10.64	2.78	3	14		
	Scorer 3	33	10.48	2.71	3	14		
IEP Complementary Components	Scorer 1	33	13.15	2.06	6	15	0.063	0.939
	Scorer 2	33	13.03	2.10	6	15		
	Scorer 3	33	12.97	2.20	6	16		
Individualised Education Programme Quality Evaluation Scale	Scorer 1	33	23.88	4.48	10	29	0.073	0.929
	Scorer 2	33	23.67	4.51	10	29		
	Scorer 3	33	23.45	4.50	10	29		

**Table 4:** Correlations between students’ scores on the IEP Quality Assessment Scale and post-test scores on the IEP Academic Achievement Test and IEP Efficacy Scale of the experimental group.

			IEP The Academic Achievement Test	Before IEP Preparation	IEP Preparation Phase	Family Participation in the IEP	Implementation of the IEP and Evolution	For Teachers IEP Efficiency Scale
Scorer I	IEP Instructional Components	r	0.182	−0.197	−0.157	−0.254	0.064	−0.175
		*p*	0.311	0.273	0.383	0.154	0.724	0.330
		N	33	33	33	33	33	33
	IEP Complementary Components	r	0.140	0.119	0.120	0.016	0.199	0.128
		*p*	0.437	0.509	0.505	0.928	0.268	0.478
		N	33	33	33	33	33	33
	Individualised Education Programme Quality Evaluation Scale	r	0.162	−0.078	−0.043	−0.165	0.134	−0.062
		*p*	0.369	0.665	0.810	0.359	0.457	0.733
		N	33	33	33	33	33	33
Scorer II	IEP Instructional Components	r	0.170	−0.100	−0.149	−0.257	0.130	−0.093
		*p*	0.344	0.579	0.409	0.149	0.470	0.607
		N	33	33	33	33	33	33
	IEP Complementary Components	r	0.134	0.081	0.153	0.084	0.126	0.101
		*p*	0.458	0.654	0.395	0.641	0.485	0.575
		N	33	33	33	33	33	33
	Individualised Education Programme Quality Evaluation Scale	r	0.167	−0.013	−0.001	−0.111	0.148	0.002
		*p*	0.352	0.942	0.995	0.539	0.410	0.990
		N	33	33	33	33	33	33
Scorer III	IEP Instructional Components	r	0.309	−0.101	−0.076	−0.189	0.133	−0.066
		*p*	0.080	0.576	0.674	0.291	0.459	0.717
		N	33	33	33	33	33	33
	IEP Complementary Components	r	0.030	0.051	0.046	0.119	0.105	0.064
		*p*	0.866	0.777	0.801	0.509	0.560	0.723
		N	33	33	33	33	33	33
	Individualised Education Programme Quality Evaluation Scale	r	0.214	−0.029	−0.020	−0.038	0.136	−0.003
		*p*	0.232	0.874	0.912	0.832	0.451	0.985
		N	33	33	33	33	33	33

## Data Availability

The datasets used and/or analysed during the current study are available from the corresponding author on reasonable request.
